# Medical Opinion and Movement

**Published:** 1908-01-04

**Authors:** 


### Medical Opinion and Movement.

Diet is one of those subjects which are always open for discussion. It is a subject upon which every physician claims a right to dogmatise in his own peculiar way, because in our present state of knowledge of the physiological functions of digestion we are unable to offer definite proof of the various orthodox tenets of the profession in regard to the relations between the different foodstuffs and certain pathological conditions. Dr. Charles J. Macalister, of Liverpool University, has recently delivered some interesting remarks on the "Personal factor in diet." His views might be summed up in the old adage, that what is one man's meat is another man's poison. He makes, however, one suggestion, which is especially worthy of consideration. He points out that whereas man as a whole is by nature, as he believes, omnivorous, there are certain classes of individuals whose ability to metabolise some one food stuff, carbohydrate or animal proteid, is limited. He suggests that the explanation of these idiosyncrasies is found in the processes of evolution, and that they are due to a reversion of the individual to some ancestral type of a herbivorous or carnivorous nature. It would be interesting to discover whether such variations from the normal in regard to diet are accompanied by other probable "reversions," such as polymastia, polythelia, and well-developed appendices vermiformes.

The open method of administering ether appears to be growing in favour among anæsthetists, and current medical literature contains several laudatory notices by exponents of the method. The apparatus and technique are very simple. The mask should be rather larger than the ordinary Schimmelbusch's, and should fit the face closely. It is covered with about sixteen layers of sterilized gauze. An ordinary 6 oz. drop bottle is fitted with a plain cork with two grooves, one larger than the other. A strand of gauze passes through the larger one, allowing the ether to drop out by capillary action. The smaller one admits air. The ether must never be poured on to the mask. If this be done some of it generally gets into the patient's mouth and sets up coughing. The disadvantages of the method are that it is often found difficult to induce anæsthesia in strong plethoric or alcoholic subjects, and that more ether is consumed than by the closed method. On the other hand, it is claimed for the method that it is surgically clean. The mask can be boiled and sterilized gauze used. The cyanosis, violent respiratory movements, and excessive secretion of mucus are greatly diminished. There is less shock than with chloroform, and there is less sickness than with ether from a closed apparatus. The difficulty of inducing anæsthesia can be overcome by commencing with a mixture of 1 part chloroform and 3 parts ether.

The rheumatic micrococcus is not often recognised as a cause of pneumonia and pleurisy, although these conditions are recognised by several authors as complications in the course of acute rheumatism. It is therefore interesting to note that within the last few weeks the diagnosis of rheumatic pneumonia has been made in no fewer than five cases. Apart from the physical signs of the chest, which in each case showed consolidation of the lungs, and also a variable amount of pleurisy and pleuritic effusion, the special points on which the diagnosis of rheumatic pneumonia rested were: that the disease was accompanied by marked pains in the joints, there was no rusty sputum, and there was an absence of the usual respiratory distress and general toxic symptoms. In one case, a girl aged 16, there was evidence of old aortic and mitral disease due to two previous attacks of acute rheumatism. According to Osler, Stephen Mackenzie found 9.94 per cent. of pneumonia and pleurisy in 3,433 cases of acute rheumatism, though in the records of Guy's Hospital from 1888 to 1898 there were only six cases of rheumatic pneumonia, or considerably less than 1 per cent. Unfortunately it does not appear that any effort was made to isolate the rheumatic micrococcus from these five recent cases. A further knowledge of the bacteriology of acute rheumatism will no doubt establish the diagnosis of these cases on a firmer basis.

American enterprise manifests itself in surgery as in other spheres of usefulness. In a recent article by Dr. Charles C. Miller, of Chicago, the "cosmetic surgery of the face" is promised a great future. Dr. Miller appears to have given a great deal of attention to the subject, and after working out the relations between the different lines of expression and the muscles which, by their continued contraction, give rise to these lines, he has devised methods of operation by which the various muscles may be divided, and the lines thereby more or less obliterated. Thus the transverse wrinkles of the forehead are, of course, due to contraction of minute fibres of the occipito-frontalis, and these he divides by subcutaneous transverse section by means of a small, sharp hook with a cutting edge. In most cases the corrugator supercilii muscles must also be divided. For the eradication of crow's feet he performs a canthotomy, and then completes the division of the orbicularis palpebrarum. The treatment of wrinkles about the mouth is on similar lines, but the different points for insertion of the hook and the particular fibres to be divided vary considerably according to the nature of the case, and a large amount of individual skill and experience are necessary for the purpose. Local anæsthesia is recommended for these operations. Dr. Miller is careful not to promise entire obliteration of the lines of age and a complete rejuvenation of the countenance, but even with this precaution the occupation sounds hazardous. It is easy to imagine that in any but very skilful hands such operations might yield as a result the opposite of what was intended.

